# Facile construction of a highly sensitive DNA biosensor by *in-situ* assembly of electro-active tags on hairpin-structured probe fragment

**DOI:** 10.1038/srep22441

**Published:** 2016-03-02

**Authors:** Qingxiang Wang, Feng Gao, Jiancong Ni, Xiaolei Liao, Xuan Zhang, Zhenyu Lin

**Affiliations:** 1 College of Chemistry and Environment, Fujian Provincial Key Laboratory of Modern Analytical Science and Separation Technology, Minnan Normal University, Zhangzhou 363000, P. R. China; 2 Ministry of Education Key Laboratory of Analysis and Detection for Food Safety, Fujian Provincial Key Laboratory of Analysis and Detection for Food Safety, Fuzhou University, Fuzhou 350116, China

## Abstract

An ultrasensitive DNA biosensor has been developed through *in-situ* labeling of electroactive melamine-Cu^2+^ complex (Mel-Cu^2+^) on the end of hairpin-like probe using gold nanoparticles (AuNPs) as the signal amplification platform. The 3′-thiolated hairpin-like probe was first immobilized to the gold electrode surface by the Au-S bond. The AuNPs were then tethered on the free 5′-end of the immobilized probe via the special affinity between Au and the modified -NH_2_. Followed by, the Mel and Cu^2+^ were assembled on the AuNPs surface through Au-N bond and Cu^2+^-N bond, respectively. Due to the surface area and electrocatalytic effects of the AuNPs, the loading amount and electron transfer kinetic of the Mel-Cu^2+^ were enhanced greatly, resulting in significantly enhanced electrochemical response of the developed biosensor. Compared with the synthesis process of conventional electroactive probe DNA accomplished by homogeneous method, the method presented in this work is more reagent- and time-saving. The proposed biosensor showed high selectivity, wide linear range and low detection limit. This novel strategy could also be extended to the other bioanalysis platforms such as immunosensors and aptasensors.

During the past decades, lots of effort has been made to exploit DNA detection technologies due to their widespread application in the fields of clinical diagnosis, environmental control, food safety and forensic analysis^1^,^2^,^3^,^4^,^5^. As an alternative to the traditional methods such as fluorescence *in*-*situ* hybridization^6^, flow cytometry^7^ and real-time quantitative reverse transcription PCR^8^, the electrochemical approach receives increasing attention since it has predominant advantages of low cost, easy operation, excellent compatibility with micro-fabrication^9^,^10^. Most of the developed DNA electrochemical biosensors were fabricated based on the linear DNA probes and the external hybridization indicators^11^,^12^,^13^. Notwithstanding, these biosensors have high sensitivity to target DNA, the low selectivity, large background response from the non-specifically bound indicators, and complicated indicators binding/rinsing process upon each hybridization step limit their practical application.

In order to overcome these disadvantages, novel sensing strategies relying on hairpin-structured DNA probe have been developed^14^,^15^,^16^,^17^,^18^,^19^. A typical hairpin-structured DNA probe in electrochemical biosensor is a single-stranded oligonucleotide consisted of two short complementary sequences at two ends (stem part) and a free sequence complementary to the target in the middle (loop part), which shows a typical hairpin-like stem-loop structure at its natural state. When the DNA probe is immobilized at an electrode surface and hybridized with the target DNA through the loop part, the stem-loop structure will be opened and transferred to rigid DNA duplex, through which the electroactive tags such as ferrocene (Fc)^14^,^15^,^16^ and methylene blue (MTB)^17^,^18^,^19^ that pre-modified at the other terminal of hairpin-structured DNA will be driven away from the electrode surface. As a consequence, the signal will be attenuated. Because the signal variation in these MB-based biosensors was strictly executed by the thermodynamically driven conformation change upon hybridization of loop part sequence with the complementary strand, outstanding hybridization selectivity, even distinguishing to single nucleotide polymorphism could be achieved.

However, most of the reported hairpin DNA-based biosensors have the following crucial defects. First, almost all the reported electroactive hairpin DNA strands are achieved through a single-point coupling reaction, *i.e.*, one hairpin DNA strand is only modified with one electroactive tag (MTB or Fc) at the probe end^14^,^15^,^16^,^17^,^18^,^19^. As a result, the signal yield as well as analysis sensitivity of these biosensors is limited, which can not satisfied the practical requirement in bioanalysis because the DNA levels in physiological samples are often very low. Secondly, the applied signal molecules, to the best of our knowledge, are only confined to Fc^14^,^15^,^16^ and MTB^17^,^18^,^19^. Although, both of them have excellent electrochemical response and are widely used as probes in electrochemical analysis, the relative insufficiency in types of electrochemical tags hampers the development of the versatile chips for high-throughput detection of multiple analytes. Thirdly, the synthesis of the redox-acive hairpin DNA strands are usually involved complicated procedures including the succinimide ester-based reaction between electroactive labels and the end-modified amino group at the probe, and the high-performance liquid chromatography (HPLC)-based separation process, which is not only time-consuming, but also labor-intensive and reagent-cost. Although the common electrochemical probes are now commercially available, the price of these probes is still relatively high in comparison with the bare DNA probes. Therefore it is still a great challenge to design and explore novel hairpin DNA-based biosensors with low cost, easy preparation process and high sensitivity.

Against this background, we reported here a novel hairpin DNA-based DNA biosensor based on *in*-*situ* assembly of signal amplification platform (gold nanoparticle, AuNPs) and new electroactive tags (melamine-copper ion complex, Mel-Cu^2+^) on the surface-confined hairpin structured DNA (Fig. 1). Mel is a cheap and highly stable six-member ring organic compound. As a trimer of cyanamide with a 1,3,5-triazine skeleton, Mel exhibits great chelating ability to many transition metal ions^20^,^21^,^22^. Recently, Zhu *et al.*^22^ reported that Mel could form stable compound with Cu^2+^ through coordination with the nitrogen atom on triazine ring, and meanwhile excellent electrochemical response from the redox of Cu^2+^/Cu^+^ couple was obtained. In addition, Mel has three free amino groups, which is favorable for its derivation with other functional groups^23^,^24^,^25^. Therefore the Mel-Cu^2+^ was chosen as an electroactive tag to graft with the probe DNA. Moreover, as an alternative to the traditional homogeneous solution-based synthesis approach, the step-by-step *in-situ* assembly of signal tags on the pre-immobilized hairpin DNA probe was applied for the fabrication of the electrochemical sensing interface, which greatly simplified the synthesis and purification process of the electroactive DNA probe. On the other hand, in order to increase the loading amount and electron transfer kinetic of the electroactive tags (Mel-Cu^2+^) on sensing interface, the highly conductive AuNPs were applied as the loading platform of the Mel-Cu^2+^ tags. The analytical assays show that the newly developed biosensor not only remains the inherent high selectivity of MB probe, but also substantially lowers the detection limit. Such a strategy is also promising to be extended to the versatile, robust aptasensors and high-throughput biochips.

## Results and Discussion

### ATR-IR and electrochemical characterization of Mel-AuNPs/apDNA/MCH/AuE

**Figure 2:**
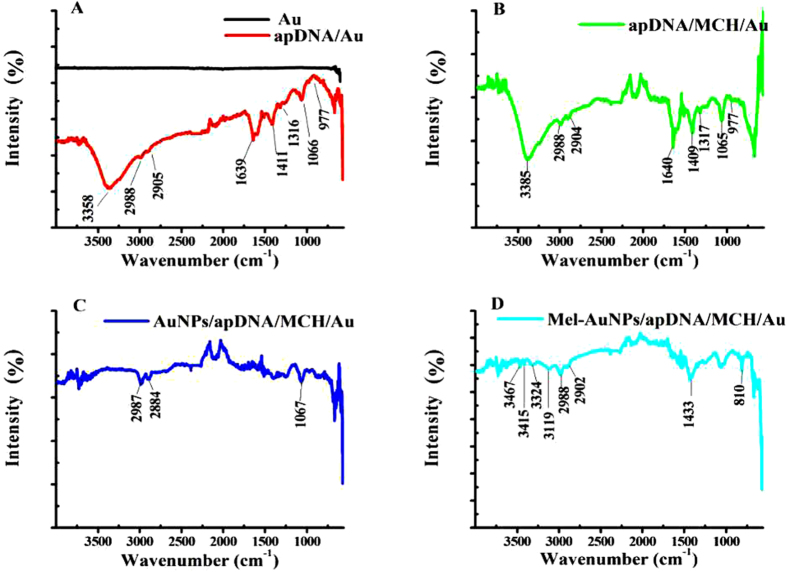
ATR-FTIR spectra of bare AuE and apDNA/AuE (**A**), apDNA/MCH/AuE (**B**), AuNPs/apDNA/MCH/AuE (**C**) and Mel-AuNPs/apDNA/MCH/AuE (**D**).

**Figure 3:**
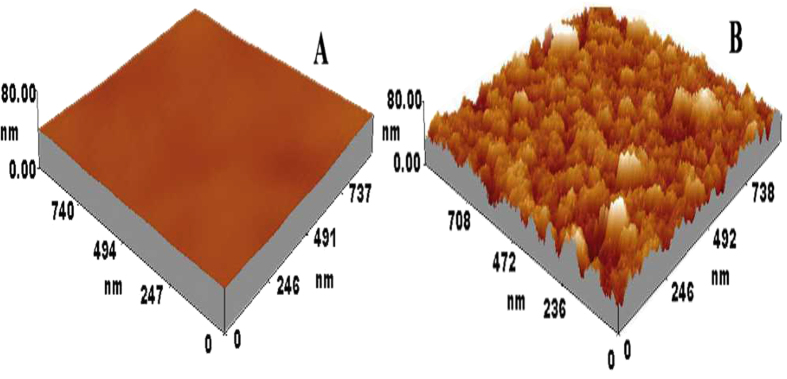
Three-dimensional AFM images of apDNA/MCH/AuE before (**A**) and after assembly with AuNPs (**B**).

ATR-FTIR is a convenient non-destructive technology to probe the adsorption of biological or organic molecules on a solid surface^26^,^27^. Therefore, the fabrication process was first characterized by the ATR-FTIR, and the results are displayed in Fig. 2. It was observed that the bare gold disk electrode (AuE) did not produce any significant features in the spectra (Fig. 2A). In the case of apDNA/AuE (Fig. 2A), some bands corresponding to the characteristic peaks of DNA were recorded^28^,^29^. The broad characteristic band in the range of 3650 ~ 3000 cm^−1^ could be assigned to N-H stretching vibrations arising from −NH_2_ in apDNA structure, and the bands at around 2988 and 2905 cm^−1^ could be assigned to the asymmetric and symmetric vibrations of C-H, respectively, arising from the −CH_2_- groups in sugar-phosphate backbone. The band at 1639 cm^−1^ was ascribed to the vibration of exocyclic –NH_2_. The bands localized at 1411 and 1316 cm^−1^ were to the base-sugar entities. The 1066 and 977 cm^−1^ bands are to the P-O stretching vibration of P-O-C groups. When apDNA/AuE was further immobilized with MCH (Fig. 2B), it is found that all the bands did not displayed significant changes. This was because all the absorption peaks corresponding to the characteristic groups of MCH masked by the absorption of DNA since these groups were also present in DNA. After the electrode was further grafted with AuNPs (Fig. 2C), it was found that the obvious and broad peak around 3358 cm^−1^ was absolutely disappeared. This could be explained by the interaction of the –NH_2_ modified on the 5′-end of apDNA with AuNPs, which limited the vibration of N-H bond. The assembly process of AuNPs on the electrode surface was also investigated by the three-dimensional atomic force microscopy (Fig. 3), which has been used extensively to probe the nanoscopic structure of the modified surface due to its high resolution^30^. As seen, some wavelike peaks were observed on apDNA/MCH/AuE (Fig. 3A), which could be ascribed to the immobilized DNA on the electrode surface. However after assembly with AuNPs, the three-dimensional changed greatly and some discrete hills were clearly observed (Fig. 3A). This confirmed that the AuNPs had been successfully grafted on the electrode surface.

In addition, when Mel was assembled (Fig. 2D), two new peaks at 3467 cm^−1^, 3324 cm^−1^ corresponding to absorption of N-H bond of Mel were observed in ATR-FTIR. From the figure, one can also observe that two new peaks appeared at 1433 cm^−1^ and 810 cm^−1^, which can be assigned to the C-N vibration and the triazine ring vibration, respectively. This also indicated that Mel molecules had been attached on the electrode surface. The fabrication process of the biosensor was further characterized by cyclic voltammetry (CV) and electrochemical impedance spectroscopy (EIS) using 1.0 mM [Fe(CN)_6_]^3−/4−^ with 0.1 M KCl as the electro-active probe, which also suggested that the biosensor has been successfully fabricated. The results and the detailed discussion were showed in Supporting Information (Fig. S2).

### Coordination assembly of Cu^2+^ on Mel/AuNPs/apDNA/MCH/AuE and its electrochemical behaviors

**Figure 4:**
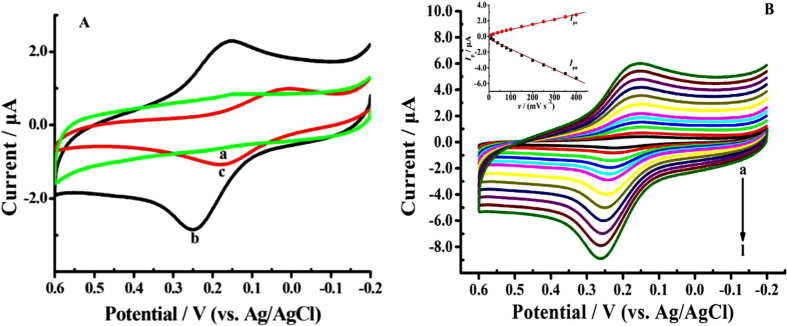
(**A**) Cyclic voltammograms of Mel-AuNPs/apDNA/MCH/AuE (a), Cu^2+^-Mel-AuNPs/apDNA/MCH/AuE (b) and Cu^2+^-Mel/cpDNA/MCH/AuE (c) in 0.1 M phosphate buffer containing 0.1 M NaClO_4_ and 0.5 M NaCl (pH 7.0). Scan rate: 100 mV s^−1^. (**B**) Cyclic voltammograms of Cu^2+^-Mel-AuNPs/apDNA/MCH/AuE with different scan rate ((a–l): 10, 20, 40, 60, 80, 100, 150, 200, 250, 300, 350, 400 mV s^−1^) and the relationship of peak currents (*I*_p_) with the scan rate (*v*) (Inset).

Figure 4A shows the CVs of Mel-AuNPs/apDNA/MCH/AuE before (curve a) and after (curve b) incubation in Cu(NO_3_)_2_ solution for 30 min. Obviously, there was not any Faradic electrochemical response for Mel-AuNPs/apDNA/MCH/AuE in supporting electrolyte of 0.1 M phosphate-buffered saline solution(PBS, pH 7.0) with 0.1 M NaClO_4_ and 0.5 M NaCl, which suggested that electrode of Mel-AuNPs/apDNA/MCH/AuE were electro-inactive in the test solution. However, after Mel-AuNPs/apDNA/MCH/AuE was immersed in Cu^2+^ solution for 30 min under gentle shaking and then tested in the above mentioned electrolyte, a pair of well-defined redox peaks was appeared at +0.26 V and +0.13 V, respectively, which was in good accordance with the electron transfer process of the Cu^2+^/Cu^+^ couple as reported in literature^23^. This suggested that the electro-active Cu^2+^ had been successfully adsorbed on the electrode surface. Figure 4B shows the CVs of Cu^2+^-Mel-AuNPs/apDNA/MCH/AuE at various scan rates. It was found that both the oxidation peak currents (*I*_pa_) and reduction peak currents (*I*_pc_) for Cu^+^/Cu^2+^ couple presented good linear relationships with the scan rates (*v*) in the range from 10 to 400 mV s^−1^ (inset of Fig. 4B): *I*_pa_(*μ*A) = −0.336 − 0.0129 *v* (mV s^−1^), and *I*_pc_(*μ*A) = 0.210 + 0.0065 *v* (mV s^−1^), with the correlation coefficients (*r*) of 0.997 and 0.998, respectively. This demonstrated that the oxidation and reduction processes of the copper ions were controlled by an adsorption process^31^, which also confirmed that the Cu^2+^ had been loaded on the electrode surface.

In addition, in order to prove the signal-amplification effect of AuNPs, a control biosensor based on assembly of Mel and Cu^2+^ on a 5′-COOH grafted DNA (cpDNA) modified AuE without using AuNPs as the loading platform was fabricated. The detailed preparation procedure was presented in Supporting Information. The electrochemical response of Cu^2+^-Mel/cpDNA/MCH/AuE was displayed as curve c in Fig. 4A. Clearly, in the absence of AuNPs, only a pair of weak redox peaks was observed on this electrode. Furthermore, the peak-to-peak separation (Δ*E*_p_) of Cu^2+^-Mel/cpDNA/MCH/AuE (curve c) was measured to be about 0.18 V, which was dramatically larger than that of Cu^2+^-Mel-AuNPs/apDNA/MCH/AuE (curve b).

In order to investigate the signal-amplification mechanism by AuNPs, the surface density (*Г*) and electron transfer rate constant (*k*_s_) of Cu^2+^-Mel tags on these two electrodes were calculated and compared. Primarily, from the CV curves of Mel-AuNPs/apDNA/MCH/AuE (curve b) and Cu^2+^-Mel/cpDNA/MCH/AuE (curve c) as showed in Fig. 4A, the electronic quantities (*Q*) of Cu^2+^-Mel-AuNPs/apDNA/MCH/AuE and Cu^2+^-Mel/cpDNA/MCH/AuE were determined to be 1.84 *μ*C and 0.46 *μ*C. Then the surface densities (*Г*) of Cu^2+^ was calculated according to the following law^32^:


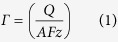


where *F* the Faraday constant, *A* the electrode surface erea, and *z* the number of electron transferred per molecule. Thus from the values of *Q* given above, the Cu^2+^ densities on Cu^2+^-Mel-AuNPs/apDNA/MCH/AuE and Cu^2+^-Mel/cpDNA/MCH/AuE were yielded to be 0.62 nmol/cm^2^ and 0.15 nmol/cm^2^. This demonstrated that the loading amount of electroactive Cu^2+^ ions on the AuNPs-assembled electrode was about 4 times higher than that without AuNPs.

Furthermore, according to the scan rate variation experiment, the electron-transfer rate constants (*k*_s_) of Cu^2+^ on these two electrodes were determined. For Cu^2+^-Mel-AuNPs/apDNA/MCH/AuE, the redox peak potentials changed slightly and all the Δ*E*_p_ values were smaller than 200/*n* mV on the tested scan rate range, so the value of *k*_s_ was obtained by the following Equation (2)^31^,^33^:


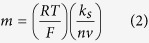


where *n* is the electron transfer number for Cu^2+^/Cu^+^, *R* the universal gas constant , *T* Kelvin temperature, *v* the scan rate. The value *m* could be obtained by adjustment of the curve Δ*E*_p_ = *f*(m^−1^) for the electron transfer coefficient (*α* = 0.5)^31^,^33^. Thus the value of *k*_s_ was determined to be 1.36 s^−1^ based on Equation (2).

However for Cu^2+^-Mel/cpDNA/MCH/AuE, it was found that the redox peak potentials shifted obviously with the increase of scan rate, and at the higher scan rate, the Δ*E*_p_ values exceeded 200/*n* mV (Fig. S3-A in Supporting Information), so the following Equations (3)–(5), 4, 5 were applied for the calculation of *k*_s_ value^31^:


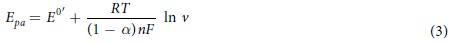



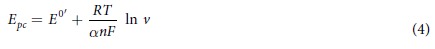



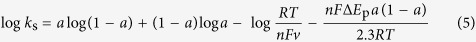


where *E*_pa_ is the anodic peak potentials, *E*^0′^ is the formal potential, *n*, *R*, *T*, and *F* have the same meanings in Equation (2). From the relationships of *E*_pa_ and *E*_pc_ versus ln *v* as displayed in Fig. S3-B in Supporting Information, the value of *k*_s_ was determined to be 0.19 s^−1^. Obviously, the *k*_s_ value of Cu^2+^-Mel-AuNPs/apDNA/MCH/AuE was extremely larger than that at Cu^2+^-Mel/cpDNA/MCH/AuE, confirming that assembled AuNPs accelerated the electron transfer kinetic of the electro-active tag. Therefore the intense electrochemical response of Cu^2+^-Mel-AuNPs/apDNA/MCH/AuE could be ascribed to the synergic effect of large loading amount of Cu^2+^-Mel and enhanced electron-transfer kinetic induced by AuNPs.

### Optimization of the experimental conditions

**Figure 5:**
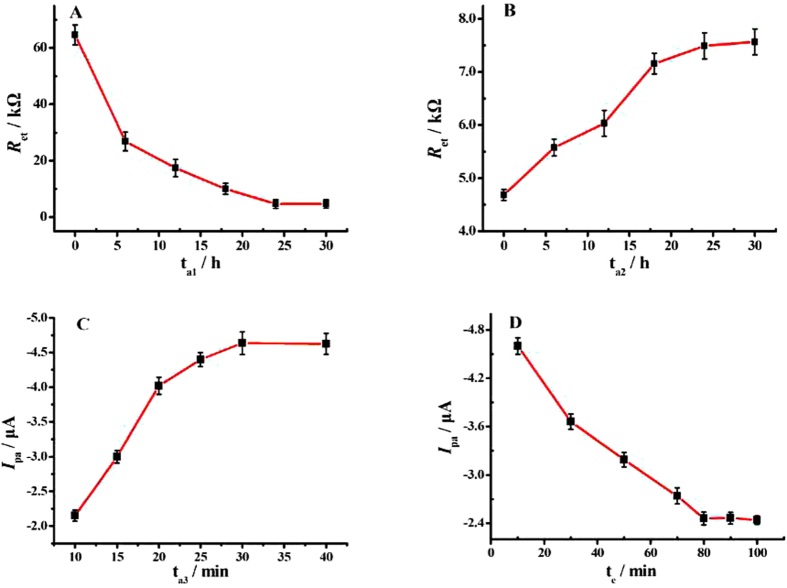
(**A**) Effects of AuNPs accumulation time (*t*_a1_) and (**B**) Mel assembly time (t_a2_) on the values of the *R*_et_. (**C**) Relationship of oxidation peak currents (*I*_pa_) with the accumulation time (t_a3_) of Mel-AuNPs/apDNA/MCH/AuE in 1.0 mM Cu(NO_3_)_2_ solution. (**D**) Relationship of oxidation peak currents (*I*_pa_) with the elution time (*t*_e_) of Cu^2+^-Mel-AuNPs/apDNA/MCH/AuE in 25.0 mM NaCl with 10.0 mM PBS (pH 7.0).

To achieve the optimal analytical performance of the biosensor, several experimental conditions had been optimized. The assembly time of AuNPs was first optimized through EIS test. As displayed in Fig. 5A, the values of *R*_et_ gradually decreased with the increase of the accumulation time (*t*_a1_) of AuNPs on apDNA/MCH/AuE, and then hardly changed after 24 h due to the adsorption saturation. Therefore, we chose 24 h as the optimal accumulation time for AuNPs in the experiments. Figure 5B displays that the values of *R*_et_ increased with the increase of the assembly time (*t*_a2_) of Mel on AuNPs/apDNA/MCH/AuE over the range from 0 to 24 h and then leveled off, which indicated that the ideal assembly time for loading the Mel was also 24 h.

The effect of coordination time of Mel-AuNPs/apDNA/MCH/AuE with Cu^2+^ on the electrochemical signal of the biosensor was investigated. Figure 5C depicts the relationship of oxidation peak currents (*I*_pa_) versus the accumulation time (t_a3_) of Cu^2+^ on Mel-AuNPs/apDNA/MCH/AuE. It was observed that the peak current value corresponding to the Cu^2+^/Cu^+^ couple enhanced with the increase of t_a3_, and then become constant after 30 min, indicating that the binding of Cu^2+^ to the electrode surface reached equilibrium at 30 min.

In addition, in order to preclude the non-specific absorption of Cu^2+^ on the electrode surface, the mixture of 25.0 mM NaCl and 10.0 mM PBS (pH 7.0) was applied as the eluting solution to remove the un-coordinated Cu^2+^. Figure 5D shows the relationship of oxidation peak currents (*I*_pa_) versus the elution time (*t*_e_), from which it could be found that the *I*_pa_ values gradually attenuated with the prolonging of *t*_e_. When the time was upon 80 min, the response signal did not decreased anymore, suggesting that the loosely absorbed Cu^2+^ had been removed from the biosensor surface. Therefore, 80 min was chosen as the optimal elution time.

### Analytical performances of the biosensor

**Figure 6:**
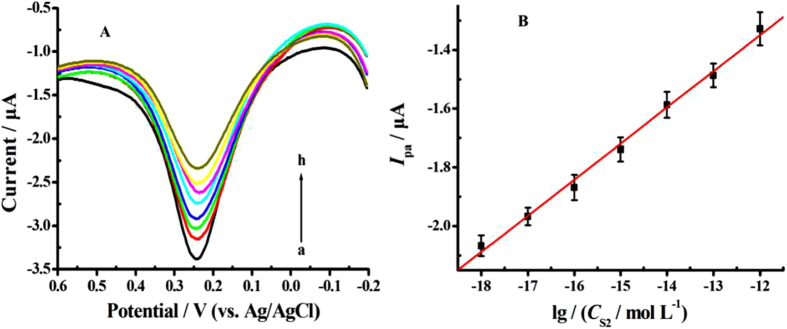
(**A**) DPVs of the biosensor hybridized with increasing concentrations of complementary DNA (S2): (a) 0 M; (b) 1.0 × 10^−18^ M; (c) 1.0 × 10^−17^ M; (d) 1.0 × 10^−16^ M; (e) 1.0 × 10^−15^ M; (f) 1.0 × 10^−14^ M; (g) 1.0 × 10^−13^ M; (h) 1.0 × 10^−12^ M. (**B**) The plot of peak currents(*I*_pa_) versus the logarithm of the target DNA concentration (lg *C*_S2_).

Under the optimum conditions, the analytical performance of the developed biosensor was evaluated by hybridization selectivity and sensitivity experiments. The sensitivity and dynamic range of the developed DNA biosensor was investigated by hybridizing the biosensor (Cu^2+^-Mel-AuNPs/apDNA/MCH/AuE) with increasing concentrations of the complementary target sequence. The DPV results were showed in Fig. 6A. From the figure, it was observed that the biosensor presented a well-defined and large oxidation peak with peak current of 2.43 *μ*A, which was found to be one to two orders larger than those on the conventional hairpin DNA-based biosensors using single Fc^14^,^15^,^16^ or MTB^17^,^18^,^19^ molecule as the signal tag, further suggesting the signal-amplification effect of the proposed biosensor. In addition, it was evidenced that the oxidation peaks of biosensor decreased accordingly as the target DNA concentration increased. This could be ascribed to the change of the hairpin probes from the loop-stem structure to the rigid linear configuration that induced by the hybridization reaction; and then the *in-situ* labeled electroactive tags of Mel-Cu^2+^ were driven away from the electrode surface. Based on the variation of the oxidation peak, it was obtained that the peak currents (*I*_pa_) of Cu^2+^/Cu^+^ couple logarithmically related to the target concentration across the range from 1.0 × 10^−18^ M to 1.0 × 10^−12^ M (Fig. 6B), with a regression equation of *I*_pa_(*μ*A) = 0.127 + 0.123 lg (*C*_S2_/M), r = 0.997. The detection limit was estimated to be 1.2 × 10^−19^ M (*ca.* 15 DNA strands) based on the signal-to-noise characteristic (S/N = 3). The analytical parameters of the developed E-DNA biosensor were compared with other hairpin DNA-based biosensors. From the results as displayed in Table S1, it could be clearly seen that our biosensor had the wider linear range and the lower detection limit than the others. It could be assigned to the intense electrochemical response resulting from the highly conductive platform of AuNPs and the high loading density of the electroactive tag (Cu^2+^-Mel).

**Figure 7:**
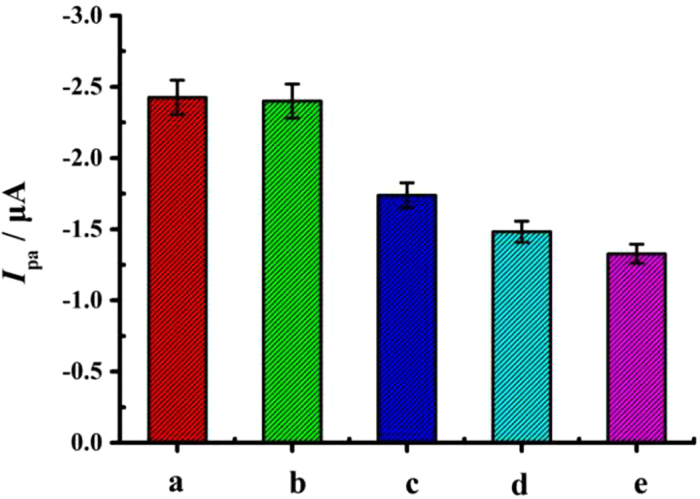
Histogram bar graph of the oxidation peak currents (*I*_pa_) in DPV of the developed biosensor without (a) and with hybridization with nDNA (b), tDNA (c), sDNA (d), and cDNA (e).

The selectivity of the electrochemical DNA biosensor was investigated through hybridization with complementary DNA (cDNA), single-base mismatched DNA (sDNA), three-base mismatched DNA (tDNA) and noncomplementary DNA (nDNA). The detailed data were displayed in Fig. 7. It was found that after hybridization with nDNA, the signal variation was negligible in comparison with the probe electrode, suggesting the hybridization reaction did not happened for the non-complementary sequences. But, upon hybridization with tDNA and sDNA, the signal decreased by about 28% and 38%, respectively. However, when the cDNA was hybridized, a substantial attenuation of 45% of the signal was produced. These results suggested that this novel DNA biosensor could be used to distinguish the cDNA from nDNA, tDNA and sDNA. We presumed that the high selectivity of the biosensor was derived from the specific stem-loop structure of the hairpin probe DNA used in this work .

### Reproductivity, regeneration and stability of the biosensor

The reproducibility and regeneration capacities play extremely important roles in practical applications for biosensors. In this work, five parallel-made DNA biosensors were used to detect 1.0 × 10^−12^ M target DNA and a relative standard deviation of 5.4% (*n* = 5) was estimated, demonstrating that the proposed biosensor had high reproducibility. The regeneration ability of this DNA biosensor was also evaluated, which was carried out by dipping the hybridized electrode in 50.0 mM NaCl at 80 °C for 20 min, followed by a rapid cooling in an ice bath for 20 min and subsequently rinsed with double-distilled water. The results showed that the developed biosensor could be repeatedly used for hybridization-denaturation-hybridization circles for 5 times, and only a decrease of 6.7% signal attenuation in DPV response was obtained. Storage of Cu^2+^-Mel-AuNPs/apDNA/MCH/AuE at 4 °C for 4 weeks resulted in a change of 2.7% in the initial DPV response, suggesting a good stability of the biosensor.

### Application in Biological Samples

To evaluate the general applicability of our proposed sensor in real sample analysis, recovery experiments were carried out by adding 1.0 pM target DNA solution into 10% (V/V) diluted healthy human serum (from Zhangzhou Affiliated Hospital of Fujian Medical University). The results showed that the recoveries were in the range of 98.5–102.7%. For each serum sample, the relative standard deviations were 6.8–8.5% with five parallel tests. These results indicated that the developed biosensor is feasible for the test of target DNA in complex samples.

## Conclusions

The conventional hairpin DNA-based biosensors have the advantages of high selectivity due to the unique stem-loop structure of the probe DNA, but the complicated preparation process and the low electrochemical signal intensity hampered the practical application of this type of biosensor. In this work, a novel and facile approach for the fabrication of the electroactive E-DNA biosensor was exploited, based on stepwise *in*-*situ* labeling of AuNPs, Mel, and Cu^2+^ on the free terminal of the hairpin probe DNA. The AuNPs were acted as a highly conductive and large surface area loading platform for the electroactive tag of Mel-Cu^2+^. With the above signal amplification strategies, the experimental results showed that the biosensor has good stability, reproductivity, regeneration and specificity, suggesting that this approach should have the potential of clinical application for detection the target DNA in biological assays. This strategy also shows great promising for the construction of versatile and robust aptasensors and high-throughput biochips.

## Methods

### Reagents and apparatus

HAuCl_4_ · H_2_O was provided by Sinopharm Chemical Reagent Co., Ltd. (China). Ethylenediaminetetraacetic acid (EDTA) and sodium citrate were purchased from Xilong Chemical Co., Ltd (China). 1-(3-Dimethylaminopropyl)-3-ethylcarbodiimide hydrochloride (EDC), *N*-Hydroxysulfosuccinimide sodium salt (sulfo-NHS), melamine (Mel), Tris (2-carboxyethyl) phosphine hydrochloride (TCEP) and 6-Hydroxy-1-hexanethiol (MCH) were purchased from Sigma-Aldrich (China). Tris (hydroxymethyl) aminomethane (Tris) and copper nitrate (Cu(NO_3_)_2_) were provided by Shanghai Jingchun Reagent Co., Ltd. (China). Phosphate-buffered saline (PBS, 0.1 M, pH 7.0) solution was prepared by mixing the stock solutions of 0.1 M NaH_2_PO_4_ and 0.1 M Na_2_HPO_4_. The gold nanoparticles (AuNPs) were synthesized according to literature^34^, and the detailed procedure was given in Supporting Information (SI). The average size of the synthesized AuNPs was estimated to be 2.1 nm by transmission electronic microscopy (TEM) as displayed in Fig. S1. All the chemicals were of analytical reagent grade and used without further purification. Double-distilled water was used throughout this experiment.

All the oligonucleotides were synthesized by Shanghai Sangon Biotechnology (China), and their sequences were listed as follows:

1. 3′-thiolated hairpin-like probe sequence with 5′-amino group (apDNA): 5′-NH_2_-(CH_2_)_6_-gcgagTCTTTGGGACCACTGTCGctcgc-(CH_2_)_6_-SH-3′;
2. 3′-thiolated hairpin-like control probe sequence with 5′-carboxyl group (cpDNA): 5′-COOH-(CH_2_)_6_-gcgagTCTTTGGGACCACTGTCGctcgc-(CH_2_)_6_-SH-3′;
3. 3′-thiolated hairpin-like control probe sequence without functional group at 5′-end (wpDNA): 5′-gcgagTCTTTGGGACCACTGTCGctcgc-(CH_2_)_6_-SH-3′;
4. Target DNA with bases complementary to the loop part of probe DNA (cDNA): 5′-CGACAGTGGTCCCAAAGA-3′;
5. Single-base mismatched DNA (sDNA): 5′-CGACAGTGGTCCCAACGA-3′;
6. Three-base mismatched DNA (tDNA): 5′-CGACAATGGCCCCAACGA-3′;
7. Non-complementary DNA (nDNA): 5′-GCATCGAGCGAGCTCGTA-3′.

DNA immobilization buffer (IB, pH 8.0) was prepared by mixing 0.1 M NaCl, 0.1 M MgCl_2_, 10.0 mM TCEP and 25.0 mM Tris-HCl. The hybridization buffer (HB) was 10.0 mM PBS (pH 7.0) with 25.0 mM NaCl. The supporting electrolyte solution for the electrochemical measurements was 0.10 M PBS (pH 7.0) with 0.1 M NaClO_4_ and 0.5 M NaCl.

Electrochemical experiments including cyclic voltammetry (CV), electrochemical impendence spectra (EIS), and differential pulse voltammetry (DPV) were measured on a CHI 650D electrochemical analyzer (China). A conventional three-electrode system, consisting of a bare or modified gold disk as working electrode, a platinum wire as auxiliary electrode and an Ag/AgCl (3.0 M KCl) as reference electrode, was used for electrochemical measurements. The geometric area of the working electrode is 3.1 × 10^−2^ cm^2^ as determined from the diameter of 2.0 mm. Attenuated total reflection infrared spectra (ATR-IR) were recorded on a NICOLET iS 10 spectrometer (USA). Atomic force microscopy (AFM) measurements were carried out on CSPM5500 (China).

### Immobilization of probe DNA on gold electrode surface

Prior to the immobilization of probe DNA, the gold disk electrode (AuE) was carefully cleaned through physical polishing, ultrasonic cleaning, electrochemical polishing as described in literature^35^. Then, the cleaned AuE was immersed into IB containing 0.1 *μ*M probe DNA (apDNA)) for 24 h to achieve probe DNA modified electrode (apDNA/AuE) through Au-S assembly chemistry. After rinsing with IB buffer and double-distilled water for several times, the modified electrode was further incubated in 1.0 mM MCH solution for 2 h to block the remaining bare regions on the electrode surface. The passivated electrode (apDNA/MCH/AuE) was then washed with IB twice. The control probes modified electrodes of cpDNA/MCH/AuE and wpDNA/MCH/AuE were prepared through the same way only replacing the apDNA with cpDNA or wpDNA.

### Step-by-step assembly of AuNPs and Mel on the free terminal of apDNA

The modified electrod was then immersed into AuNPs solution for 24 h at 4 °C. Thus the AuNPs was assembled on the terminal of hairpin-like apDNA via the affinity with the modified amine groups on 5′-end. Then the electrode was rinsed with double-distilled water for three times to remove the nonspecifically absorbed AuNPs. The obtained electrode was denoted as AuNPs/apDNA/MCH/AuE. After blow-drying the electrode surface with N_2_, the Mel was further assembled on the grafted AuNPs through immersing AuNPs/apDNA/MCH/AuE into 1.0 mM Mel aqueous solution for 24 h with gentle shaking. Then the electrode with the confined Mel molecules (Mel-AuNPs/apDNA/MCH/AuE) was achieved after extensively rinsing with double-distilled water to remove the physically absorbed Mel.

### Coordination assembly of copper ions on the sensing interface

The electroactive Cu^2+^ ions were finally attached on the sensing interface through coordination with Mel. In brief, the Mel-AuNPs/apDNA/MCH/AuE was incubated in 1.0 mM Cu(NO_3_)_2_ for 30 min under gentle stirring. Then the electrode was immersed in a mixture solution containing 25.0 mM NaCl and 10.0 mM PBS for 80 min at 37 °C to remove the Cu^2+^ electrostatically adsorbed on the phosphate backbone of probe DNA. The obtained electrode was denoted as Cu^2+^-Mel-AuNPs/apDNA/MCH/AuE.

### Hybridization and electrochemical detection of target DNA

The hybridization reaction of the biosensor was performed by immersing Cu^2+^-Mel-AuNPs/apDNA/MCH/AuE into 200 *μ*L HB containing different targets for 40 min at 37 °C. After rinsed with TE to remove the non-specifically bound DNA, the hybridized electrode was obtained.

Electrochemical characterization on the fabrication process of the biosensor was carried out in 1.0 mM [Fe(CN)_6_]^3−/4−^ solution containing 0.1 M KCl via CV and EIS. The scan range for CV was between −0.2 and +0.6 V with the scan rate of 100 mV/s. The EIS was collected at a potential of +0.207 V in the frequency range of 10^5^ ~ 1 Hz with the voltage amplitude of 5 mV. The electrochemical behaviours and the hybridization monitoring of the developed biosensor were carried out in supporting electrolyte through CV and differential pulse voltammetry (DPV). The potential range for the CV measurement was from −0.2 to +0.6 V. The DPV was recorded at an increment potential of 0.004 V, pulse amplitude of 0.05 V, pulse width of 0.05 s, sample width of 0.0167 s, pulse period of 0.2 s, and quiet time of 2 s.

## Additional Information

**How to cite this article**: Wang, Q. *et al.* Facile construction of a highly sensitive DNA biosensor by *in-situ* assembly of electro-active tags on hairpin-structured probe fragment. *Sci. Rep.* **6**, 22441; doi: 10.1038/srep22441 (2016).

## Supplementary Material

Supplementary Information

## Figures and Tables

**Figure 1:**
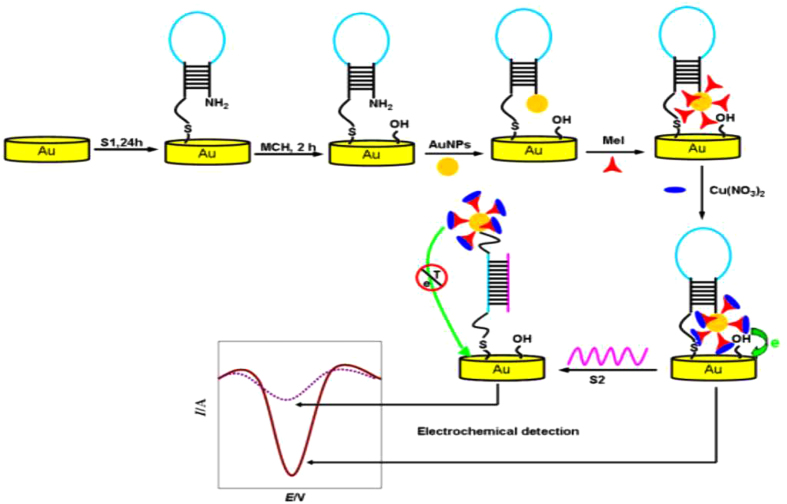
Schematic illustration for the fabrication process of the DNA biosensor using *in*-*situ* assembled AuNPs-Mel-Cu^2+^ as the signal tag.

## References

[b1] Jia Y. *et al.* Rational designed bipolar, conjugated polymer-DNA composite beacon for the sensitive detection of proteins and ions. Anal. Chem. 87, 3890–3894 (2015). 25694029 10.1021/ac504690y

[b2] Xu G., Gunson R. N., Cooper J. M. & Reboud J. Rapid ultrasonic isothermal amplification of DNA with multiplexed melting analysis-applications in the clinical diagnosis of sexually transmitted diseases. Chem. Commun. 51, 2589–2592 (2015). 10.1039/c4cc08389j 25569801

[b3] Egan S. P. *et al.* Rapid molecular detection of invasive species in ballast and harbor water by integrating environmental DNA and light transmission spectroscopy. Environ. Sci. Technol. 49, 4113–4121 (2015). 25686279 10.1021/es5058659

[b4] Zheng J. *et al.* Universal surface-enhanced raman scattering amplification detector for ultrasensitive detection of multiple target analytes. Anal. Chem. 86, 2205–2212 (2014). 24437937 10.1021/ac404004m

[b5] Abdalhai M. H. *et al.* Rapid and sensitive detection of foodborne pathogenic bacteria (staphylococcus aureus) using an electrochemical DNA genomic biosensor and its application in fresh beef. J. Agric. Food Chem. 62, 2659–12667 (2014). 10.1021/jf503914f 25474119

[b6] Lee P. K., Men Y., Wang S., He J. & Alvarez-Cohen L. Development of a fluorescence-activated cell sorting method coupled with whole genome amplification to analyze minority and trace dehalococcoides genomes in microbial communities. Environ. Sci. Technol. 49, 1585–1593 (2015). 25565465 10.1021/es503888y

[b7] Freitas C., Parreira T. M., Roseiro J., Reis A. & da Silva T. L. Selecting low-cost carbon sources for carotenoid and lipid production by the pink yeast Rhodosporidium toruloides NCYC 921 using flow cytometry. Bioresour. Technol. 158, 355–359 (2014). 24650616 10.1016/j.biortech.2014.02.071

[b8] Chen H., Zhu Z., Lu J. J. & Liu S. Charging YOYO-1 on capillary wall for online DNA intercalation and integrating this approach with multiplex PCR and bare narrow capillary-hydrodynamic chromatography for online DNA analysis. Anal. Chem. 87, 1518–1522 (2015). 25555111 10.1021/ac504257b PMC4318619

[b9] Miodek A., Mejri N., Gomgnimbou M., Sola C. & Korri-Youssoufi H. E-DNA sensor of mycobacterium tuberculosis based on electrochemical assembly of nanomaterials (MWCNTs/PPy/PAMAM). Anal. Chem. 87, 9257–9264 (2015). 26313137 10.1021/acs.analchem.5b01761

[b10] Tan Y. *et al.* Ultraselective homogeneous electrochemical biosensor for DNA species related to oral cancer based on nicking endonuclease assisted target recycling amplification. Anal. Chem. 87, 9204–9208 (2015). 26295334 10.1021/acs.analchem.5b01470

[b11] Shiddiky M. J. A., Torriero A. A. J., Zeng Z., Spiccia L. & Bond A. M. Highly selective and sensitive DNA assay based on electrocatalytic oxidation of ferrocene bearing zinc(II)-cyclen complexes with diethylamine. J. Am. Chem. Soc. 132, 10053–10063 (2010). 20597510 10.1021/ja1021365

[b12] Zheng D. *et al.* Development of a novel electrochemical DNA biosensor based on elongated hexagonal-pyramid CdS and poly-isonicotinic acid composite film. Biosens. Bioelectron. 60, 167–174 (2014). 24800680 10.1016/j.bios.2014.04.011

[b13] Liu A. & Anzai J. Use of polymeric indicator for electrochemical DNA sensors: Poly(4-vinylpyridine) derivative bearing [Os(5,6-dimethyl-1,10-phenanthroline)_2_Cl]^2^^+^. Anal. Chem. 76, 2975–2980 (2004). 15144212 10.1021/ac0303970

[b14] Fan C., Plaxco K. W. & Heeger A. J. Electrochemical interrogation of conformational changes as a reagentless method for the sequence-specific detection of DNA. PNAS 100, 9134–9137 (2003). 12867594 10.1073/pnas.1633515100 PMC170884

[b15] Chatelain G., Brisset H. & Chaix C. A thermodynamic study of ferrocene modified hairpin oligonucleotides upon duplex formation: applications to the electrochemical detection of DNA. New J. Chem. 33, 1139–1147 (2009).

[b16] Yan D. *et al.* Reagentless, ratiometric electrochemical DNA sensors with improved robustness and reproducibility. Anal. Chem. 86, 8010–8016 (2014). 25010201 10.1021/ac5025254 PMC4372097

[b17] Farjami E., Clima L., Gothelf K. & Ferapontova E. E. “Off-On” electrochemical hairpin-DNA-based genosensor for cancer diagnostics. Anal. Chem. 83, 1594–1602 (2011). 21314139 10.1021/ac1032929

[b18] He X. *et al.* A Simple, fast, and sensitive assay for the detection of DNA, thrombin, and adenosine triphosphate based on dual-hairpin DNA structure. Langmuir 29, 14328–14334 (2013). 24079405 10.1021/la403192p

[b19] Xiao Y., Lubin A. A., Baker B. R., Plaxco K. W. & Heeger A. J. Single-step electronic detection of femtomolar DNA by target-induced strand displacement in an electrode-bound duplex. PNAS 103, 16677–16680 (2006). 17065320 10.1073/pnas.0607693103 PMC1622927

[b20] Li L. *et al.* Graphene quantum dots as fluorescence probes for turn-off sensing of melamine in the presence of Hg^2+^. ACS Appl. Mater. Interfaces 6, 2858–2864 (2014). 24460139 10.1021/am405305r

[b21] Zhang Y., Chen K. & Fan H. T. Synthesis, crystal structure and catalytic property of a new Cu(I) coordination polymer constructed from melamine and azide ligands. Inorg. Chem. Commun. 38, 47–49 (2013).

[b22] Zhu H., Zhang S., Li M., Shao Y. & Zhu Z. Electrochemical sensor for melamine based on its copper complex. Chem. Commun. 46, 2259–2261 (2010). 10.1039/b924355k 20234925

[b23] Schwab M. G. *et al.* Catalyst-free preparation of melamine-based microporous polymer networks through Schiff base chemistry. J. Am. Chem. Soc. 131, 7216–7217 (2009). 19469570 10.1021/ja902116f

[b24] Grondein A. & Bélanger D. Covalent grafting of aminated compounds on Vulcan XC72R by melamine *in situ* diazotization. Carbon 50, 4335–4342 (2012).

[b25] Edwards G. A. *et al.* Melamine and melamine-formaldehyde polymers as ligands for palladium and application to suzuki-miyaura cross-coupling reactions in sustainable solvents. J. Org. Chem. 79, 2094–2104 (2014). 24533440 10.1021/jo402799t

[b26] Di Giambattista L. *et al.* New marker of tumor cell death revealed by ATR-FTIR spectroscopy. Anal. Bioanal. Chem. 399, 2771–2778 (2011). 21249341 10.1007/s00216-011-4654-7

[b27] Mircescu N. E., Oltean M., Chiş V. & Leopold N. FTIR, FT-Raman, SERS and DFT study on melamine. Vib. Spectrosc. 62, 165–171 (2012).

[b28] Stepanyugin A. V., Samijlenko S. P., Martynenko O. I. & Hovorun D. M. ATR-IR spectroscopy as applied to nucleic acid films. Spectrochim. Acta A 61, 2267–2269 (2005). 10.1016/j.saa.2004.09.019 15911421

[b29] Cui H. F. *et al.* Hairpin DNA as a biobarcode modified on gold nanoparticles for electrochemical DNA detection. Anal. Chem. 87, 1358–1365 (2015). 25530496 10.1021/ac504206n

[b30] Burt D. P., Wilson N. R., Janus U., Macpherson J. V. & Unwin P. R. *In-situ* atomic force microscopy (AFM) imaging: influence of AFM probe geometry on diffusion to microscopic surfaces. Langmuir 24, 12867–12876 (2008). 18558780 10.1021/la8003323

[b31] Laviron E. General expression of the linear potential sweep voltammogram in the case of diffusionless electrochemical systems. J. Electroanal. Chem. 101, 19–28 (1979).

[b32] Tsai W. Y., Taberna P. L. & Simon P. Electrochemical quartz crystal microbalance (EQCM) study of ion dynamics in nanoporous carbons. J. Am. Chem. Soc. 136, 8722–8728 (2014). 24869895 10.1021/ja503449w

[b33] Campos R. & Ferapontova E. E. Electrochemistry of weakly adsorbed species: Voltammetric analysis of electron transfer between gold electrodes and Ru hexaamine electrostatically interacting with DNA duplexes. Electrochim. Acta 126, 151–157 (2014).

[b34] Jana B. R., Gearheart L. & Murphy C. J. Seed-mediated growth approach for shape-controlled synthesis of spheroidal and rod-like gold nanoparticles using a surfactant template. Adv. Mater 13, 1989–1993 (2001).

[b35] Yang K. & Zhang C. Y. Improved sensitivity for the electrochemical biosensor with an adjunct probe. Anal. Chem. 82, 9500–9505 (2010). 20979391 10.1021/ac102189e

