# Supplementary material for: Facile construction of a highly sensitive DNA biosensor by in-situ assembly of electro-active tags on hairpin-structured probe fragment
Source: Sci Rep. 2016 Mar 2;6:22441. doi: 10.1038/srep22441 (PMC4773755; doi:10.1038/srep22441)
Supplement: Supplementary Information [file srep22441-s1.doc]

Supporting Information

**Facile construction of a highly sensitive DNA biosensor by *in-situ* assembly of electro-active tags on hairpin-structured probe fragment**

Qingxiang Wang^1^*, Feng Gao^1^, Jiancong Ni^1, 2^, Xiaolei Liao^1^, Xuan Zhang^1^, & Zhenyu Lin^2^

1 College of Chemistry and Environment, Fujian Provincial Key Laboratory of Modern Analytical Science and Separation Technology, Minnan Normal University, Zhangzhou 363000, P. R. China

2 Ministry of Education Key Laboratory of Analysis and Detection for Food Safety, Fujian Provincial Key Laboratory of Analysis and Detection for Food Safety, Fuzhou University, Fuzhou 350116, China

***Corresponding Author***

** E-mail:* [*axiang236@126.com*](mailto:axiang236@126.com)*. Tel: +86-596-2591445. Fax: +86-596-2520035.*

**Table of contents:**

1. **Synthesis and TEM characterization of the AuNPs**
2. **Electrochemical characterization on the fabrication of the biosensor**

**3. Fabrication process of Cu^2+^-Mel/cpDNA/MCH/AuE**

**Fig. S1.** TEM image of the synthesized AuNPs nanoparticles and the diameter distribution graph (Inset).

**Fig. S2. Figure S2**. Cyclic voltammograms (A) and electrochemical impedance spectroscopy (B) of 1.0 mM [Fe(CN)_6_]^3-/4-^ with 0.1 M KCl recorded on bare AuE (a), apDNA/AuE (b), apDNA/MCH/AuE (c), AuNPs/apDNA/MCH/AuE (d) and Mel-AuNPs/apDNA/MCH/AuE (e). Inset of B is the magnified Nyquist plots of bare AuE (a) and AuNPs/apDNA/MCH/AuE (d).

**Fig. S3.** The oxidation peak intensity of CV in 1.0 mM [Fe(CN)_6_]^3-/4-^ with 0.1 M KCl recorded on wpDNA/MCH/AuE before (a) and after assembly with AuNPs (b), and apDNA/MCH/AuE before (c) and after assembly with AuNPs (d).

**Fig. S4.** The linear relationship of the peak potential *E*_pa_ (a) and *E*_pc_ (b) versus ln *v*.

**Table S1.** Comparison of analytical performance electrochemical of DNA biosensors based on MBs with different signal tags or signal amplification strategies.

**1. Synthesis and TEM characterization of the AuNPs**

The gold nano-particles were synthesized according to the method reported by Zhang et. al.^1^ All glassware was required thoroughly cleaning with Aqua regia (HCl/HNO_3_ 3:1, V/V). 1 mM HAuCl_4_ solution was suspended in a 250 mL conical flask and heated to boiling on a stirring hot plate. Then 38.8 mM Sodium citrate was added to above HAuCl_4_ solution under constant stirring. The color was changed from yellow to pink. The solution was subsequently heated and stirred continuously for 7~8 min. After this, the solution was cooled to natural temperature under stirring. Then the solution was transferred into a clear volumetric flack, and diluted to 100 mL with DDW. This volumetric flack was kept in the refrigerator until used.

Fig. S1 shows the typical TEM image of the synthesized AnNPs. As seen, some small and uniform spherical particles were obtained. From the corresponding diameter distribution graph (inset), the average diameter of the particles was estimated to be 2.1 nm.


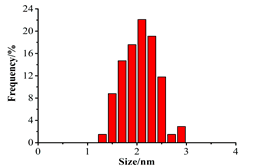

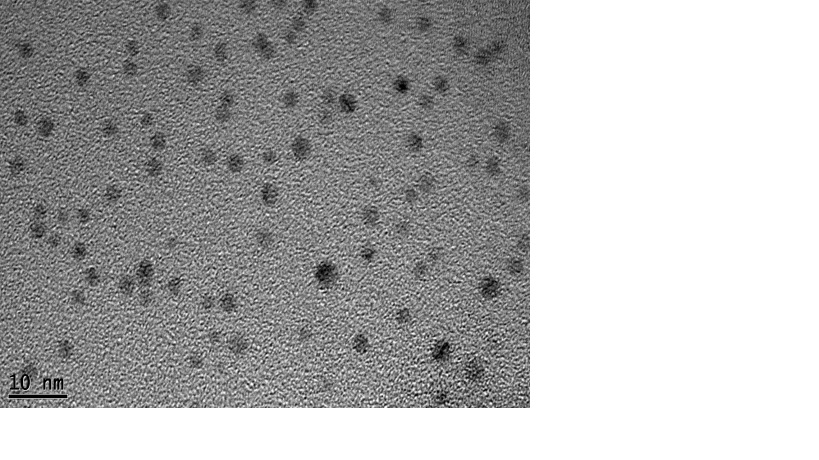


**Figure. S1** TEM image of the synthesized AuNPs nanoparticles and the diameter distribution graph (Inset).

1. **Electrochemical characterization on the fabrication of the biosensor**

The step-by-step assembly process of the modified electrode was also characterized through electrochemical method using 1.0 mM [Fe(CN)_6_]^3-/4-^ with 0.1 M KCl as the electro-active probe. The CV results were depicted in following Figure S2-A. As seen, a pair of reversible redox peak corresponding to the electron transfer of [Fe(CN)_6_]^3-/4-^ couple was observed on bare AuE (curve a). After immobilization of probe DNA and MCH on AuE sequentially, the redox peaks decreased and the peak-to-peak separation (Δ*E*_p_) increased accordingly (curve b and curve c), due to the repulsion action of negatively charged phosphate backbone on probe DNA to the negative redox probe, [Fe(CN)_6_]^3-/4-^, and the backfill of the uncovered gold surface by MCH.^2^ However, after the modified electrode of apDNA/MCH/AuE was reacted with AuNPs, the redox peak of [Fe(CN)_6_]^3-/4-^ increased significantly with the reduction of Δ*E*_p_ (curve d). This suggested that the electron transfer kinetic of [Fe(CN)_6_]^3-/4-^ on the electrode surface was greatly enhanced by the highly conductive AuNPs. In order to probe the assembly mechanism of AuNPs to the 5’-NH_2_ modified probe DNA, a control strand without 5’-NH_2_ (wpDNA) modified electrode (wpDNA/MCH/AuE) was prepared through the similar method, and its interaction with AuNPs was also characterized by CV. Figure S3 displayed the the oxidation peak intensity of [Fe(CN)_6_]^3-/4-^ recorded on wpDNA/MCH/AuE before (a) and after assembly with AuNPs (b), and apDNA/MCH/AuE before (c) and after assembly with AuNPs (d). As shown, the change of oxidation peaks of [Fe(CN)_6_]^3-/4-^ on control electrode was significantly smaller than that at apDNA/MCH/AuE. These results indicated that the free 5’-NH_2_ played an important role for grafting AuNPs as a platform, which could be ascribed to the formation of the robust Au-N bond.^3,4^ Furthermore, when the AuNPs/apDNA/MCH/AuE electrode was further incubated with Mel solution, the electrochemical response of [Fe(CN)_6_]^3-/4-^ showed a decrease (curve e), suggesting that Mel had been confined on the surface of AuNPs modified probe DNA via the Au-NH_2_ affinity, and blocked the approaching of [Fe(CN)_6_]^3-/4-^ to the surface of grafted AuNPs. EIS is a sensitive and powerful technique being able to monitor change of the interfacial properties at the electrode surface upon stepwise modification.^5-7^ In a EIS experiment, the typical Nyquist plot is composed of a semicircular portion in a high-frequency region and a linear portion in low-frequency region. The electron-transfer resistance (*R*_et_) on the electrode surface could be directly reflected by the diameter of the semicircle at the high-frequency region.

The step-by-step modification process of the sensing interface was also investigated by EIS experiments. Figure S2-B illustrates the Nyquist plots of stepwise modified electrodes in 1.0 mM [Fe(CN)_6_]^3-/4-^ with 0.1 M KCl. As shown, the bare AuE showed a very small semicircle domain with the *R*_et_ value of 345 Ω, which indicated well-behaved electron transfer ability of the bare AuE. When apDNA and MCH were immobilized on the electrode surface via Au-S, the *R*_et_ values increased to 8700 Ω and to 64630 Ω (curve b and c), respectively, suggesting a mixture film of probe DNA and MCH had been anchored on the electrode surface. After AuNPs were grafted, a dramatic drop in *R*_et_ with the value of 4685 Ω was observed, testifying that the electron transfer kinetic of [Fe(CN)_6_]^3-/4-^ was enhanced by the highly conductive AuNPs (curve d). When Mel molecules were further assembled, the *R*_et_ increased to 7491 Ω, which could be attributed to the fact that access of [Fe(CN)_6_]^3-/4-^ to the electrode surface was inhibited by the formed Mel layer (curve e). All these variation trends were in good consistence with those in CV experiments, and suggested the successful fabrication of the sensing interface.


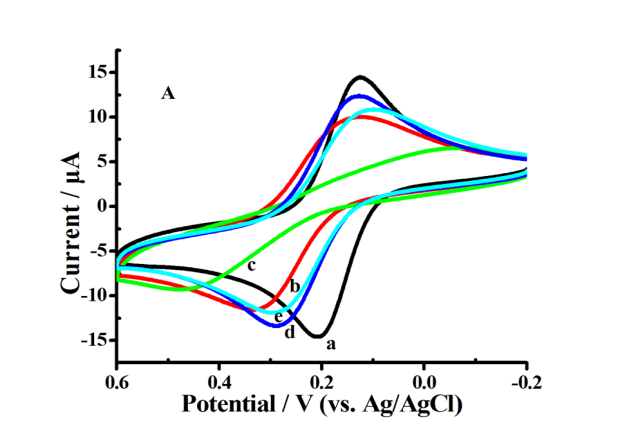

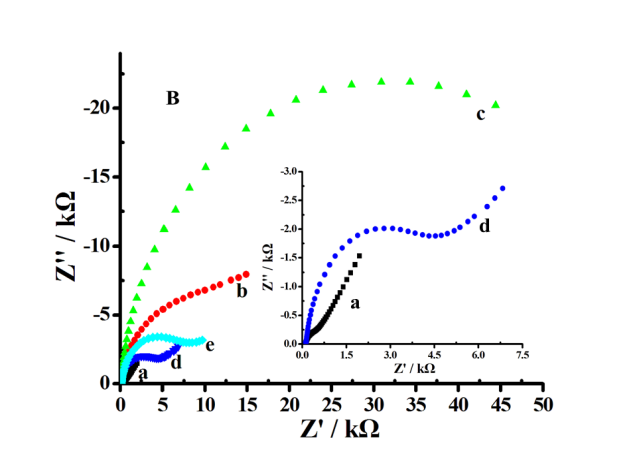


**Figure S2**. Cyclic voltammograms (A) and electrochemical impedance spectroscopy (B) of 1.0 mM [Fe(CN)_6_]^3-/4-^ with 0.1 M KCl recorded on bare AuE (a), apDNA/AuE (b), apDNA/MCH/AuE (c), AuNPs/apDNA/MCH/AuE (d) and Mel-AuNPs/apDNA/MCH/AuE (e). Inset of B is the magnified Nyquist plots of bare AuE (a) and AuNPs/apDNA/MCH/AuE (d).

**Figure. S3**. The oxidation peak intensity of CV in 1.0 mM [Fe(CN)_6_]^3-/4-^ with 0.1 M KCl recorded on wpDNA/MCH/AuE before (a) and after assembly with AuNPs (b), and apDNA/MCH/AuE before (c) and after assembly with AuNPs (d).

**Fabrication process of** **Cu^2+^-Mel/cpDNA/MCH/AuE**

The electrode cleaning, cpDNA and MCH assembly process were the same to those of apDNA/MCH/AuE as described in Experimental section. Then the cpDNA/MCH/AuE was immersed in 25 mM Tris-HCl buffer containing EDC (4mM) and NHS (8mM) for 1 h to active the –COOH group modified at the 5’-terminum at cpDNA. Then the actived cpDNA/MCH/AuE was immediately immersed in 1.0 mM Mel solution for 1 h. After removing the non-covalently bound Mel by rinsing with Tris-HCl buffer, the Mel/cpDNA/MCH/AuE was obtained. Then the electrode further was incubate into 1.0 mM Cu^2+^ for 30 min for coordination assembly, and then immersed into 25.0 mM NaCl and 10.0 mM PBS for 80 min at 37 ^o^C to remove the loosely bound Cu^2+^.


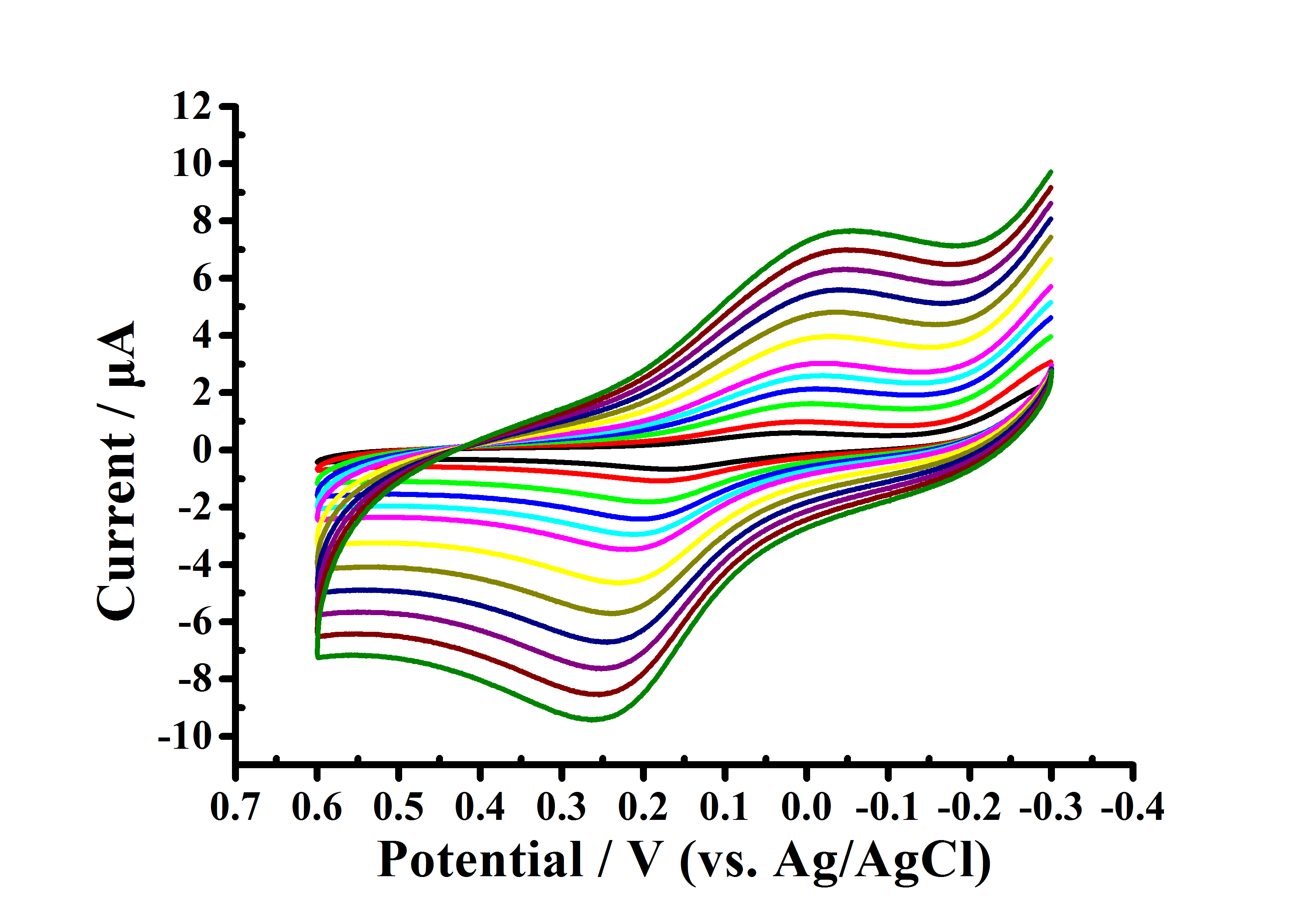

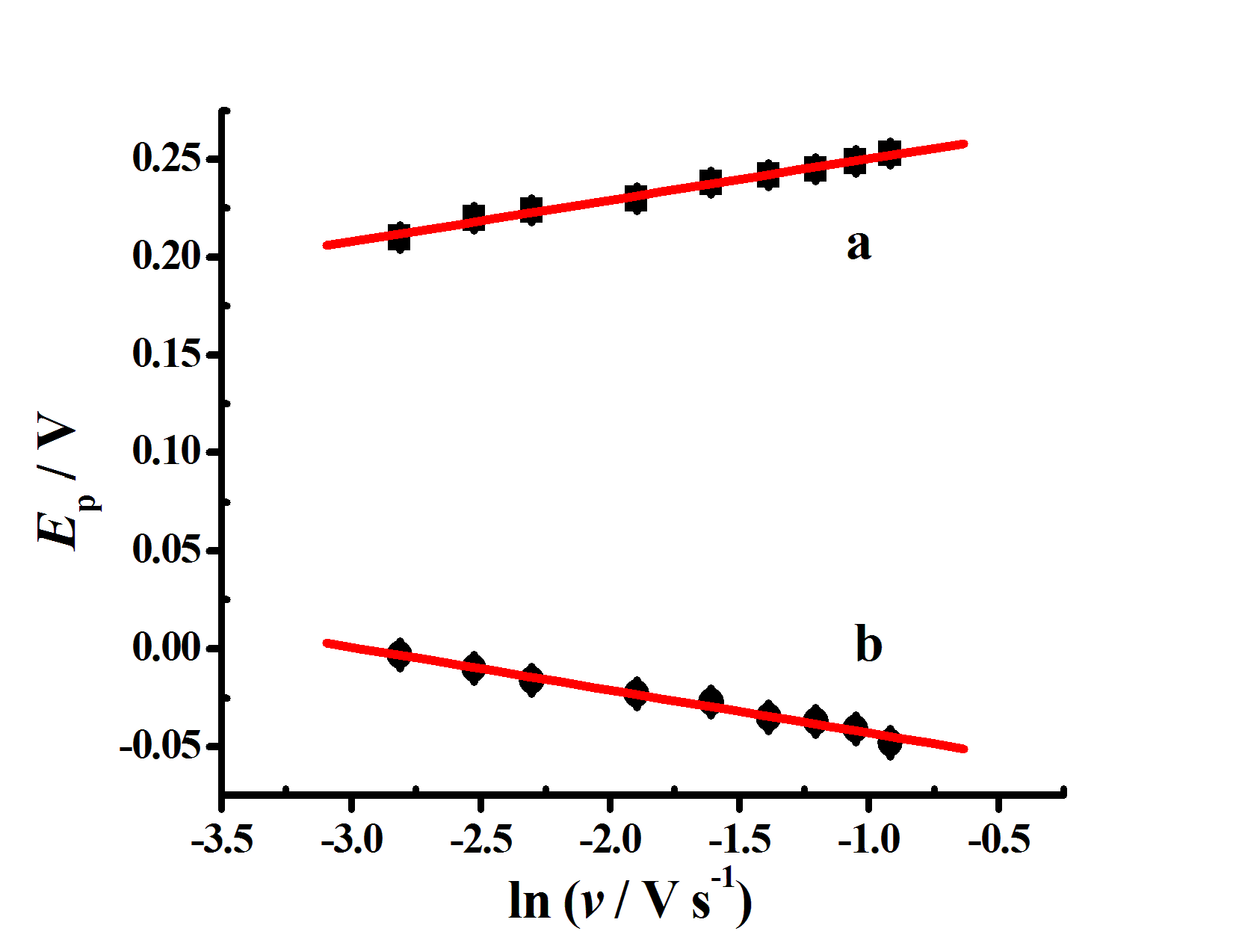


**10 mV s^-1^**

**400 mV s^-1^**

**A**

**A**

**B**

**Figure. S4.** (A) Cyclic voltammograms of Cu^2+^-Mel/cpDNA/MCH/AuE in SES with different scan rate and (B) the relationships of the peak potential *E*_pa_ (a) and *E*_pc_ (b) versus ln *v*.

**Table S1.** Comparison of analytical performance electrochemical of DNA biosensors based on hairpin-structured probe strands

| **Sensing tags** | **Methods (signal molecules)** | **Target DNA (NOB)** | **Linear ranges** | **Detection limit** | **Refs.** |
| --- | --- | --- | --- | --- | --- |
| Ferrocene | DPV (ferrocene) | Not given (27) | 30 pM -50μM | 10 pM | 8 |
| Ru(bpy)_2_(dcbpy)-NHS | ECL (Ru(bpy)_2_(dcbpy)) | *ARN 23S gene* (16) | 0.27 nM~4.0 nM | 90 pM | 9 |
| Anti-DIG-HRP | Amperometry (TMB+H_2_O_2_) | *Escherichia coli* (36) | 10 fM~10 nM | 10 fM | 10 |
| Biotin | EIS ([Fe(CN)_6_]^3-/4-^) | *Peanut allergen Ara h* 1 (25) | 1.0 fM~10 nM | 0.35 fM | 11 |
| FeTCPP@MOF | DPV (PED+ H_2_O_2_) | Not given (25) | 10 fM~10 nM | 0.48 fM | 12 |
| CdTe nanoparticals | EIS ([Fe(CN)_6_]^3-/4-^) | Not given (24) | 4.66 fM~4.66 μM | 4.7 fM | 13 |
| biotin -HRP-SA | Amperometry (HQ +H_2_O_2_) | *Peanut allergen Ara h* 1 (25) | 39.1 aM~1.25 fM | 13 aM | 14 |
| Ferrocene | DPV (four-ferrocene) | Not given (50) | 5 pM -5 nM | 3.5 pM | 15 |
| Cu-Mel-AuNPs | DPV (Cu^2+^-Mel) | CaMV35S promoter gene (18) | 1.0 aM~1.0 pM | 0.12 aM | This work |

Notes: NOB: number of the bases; DPV=differential pulse voltammetry; Ru(bpy)_2_(dcbpy)-NHS= Ruthenium bis(2,2’-bipyridine)-(2,2’-bipyridine-4,4’-dicarboxylic acid)-*N*-hydroxysuccinimide ester; anti-DIG-HRP: horseradish peroxidase-linked-anti-DIG antibody; TMB=3,3’,5,5’-tetramethylbenzidine; EIS= electrochemical impendence spectra; FeTCPP =iron(III) *meso*-5,10,15,20-tetrakis(4-carboxyphenyl) porphyrin; MOF=HKUST-1(Cu) metal-organic framework; PED=phenylenediamine; HRP-SA=streptavidin-horseradish peroxidase; HQ: hydroquinone; Mel=melamine.

**Reference**

1. Jana, B.R., Gearheart, L. & Murphy, C.J. Seed-mediated growth approach for shape-controlled synthesis of spheroidal and rod-like gold nanoparticles using a surfactant template. *Adv. Mater* **13***,* 1989-1993 (2001).
2. Elshafey, R., Siaj, M. & Zourob, M. In vitro selection, characterization, and biosensing application of high-affinity cylindrospermopsin-targeting aptamers. *Anal. Chem.* **86**, 9196-9203 (2014).
3. Zhang, X. *et al.* Colorimetric sensing of clenbuterol using gold nanoparticles in the presence of melamine. *Biosens*. *Bioelectron*. **34**, 112-117 (2012).
4. Shein, J. B., Lai, L. M., Eggers, P. K., Paddon-Row, M. N. & Gooding, J. J. Formation of efficient electron transfer pathways by adsorbing gold nanoparticles to self-assembled monolayer modified electrodes. *Langmuir* **25**, 11121-11128 (2009).
5. Veloso, A. J. et al. Electrochemical immunosensors for effective evaluation of amyloid-beta modulators on oligomeric and fibrillar aggregation processes. *Anal. Chem.* **86**, 4901-4909 (2014).
6. Dominguez-Benetton, X., Sevda, S., Vanbroekhoven, K. & Pant, D. The accurate use of impedance analysis for the study of microbial electrochemical systems. *Chem. Soc. Rev.* **41**, 7228-7246 (2012).
7. Riedel, M., Kartchemnik, J., Schöning, M. J. & Lisdat, F. Impedimetric DNA detection-steps forward to sensorial application. *Anal. Chem.* **86,** 7867-7874 (2014).
8. Fan, C., Plaxco, K. W. & Heeger, A. J. Electrochemical interrogation of conformational changes as a reagentless method for the sequence-specific detection of DNA. *PNAS* **100**, 9134-9137 (2003).
9. Zhang, J. *et al.* Electrogenerated chemiluminescence DNA biosensor based on hairpin DNA probe labeled with ruthenium complex. *Anal. Chem.* **80**, 2888-2894 (2008).
10. Liu, G. *et al.* An Enzyme-based E-DNA sensor for sequence-specific detection of femtomolar DNA targets. *J. Am. Chem. Soc.* **130**, 6820-6825 (2008).
11. Sun, X. L., Guan, L., Shan, X. H., Zhang, Y. Z. & Li, Z. J. Electrochemical detection of peanut allergen Ara h 1 using a sensitive DNA biosensor based on stem-loop probe. J. Agric. Food Chem. **60**, 10979-10984 (**2012**).
12. Ling, P. H., Lei, J. P., Zhang, L. & Ju, H. X. Porphyrin-encapsulated metal-organic frameworks as mimetic catalysts for electrochemical DNA sensing via allosteric switch of hairpin DNA. *Anal. Chem.* **87**, 3957-3963 (2015).
13. Kjällman, T. H. M., Peng, H., Soeller, C. & Travas-Sejdic, J. A CdTe nanoparticle-modified hairpin probe for direct and sensitive electrochemical detection of DNA. *Analyst* **135**, 488-494 (2010).
14. Sun, X. L. *et al.* Enzymatic amplification detection of peanut allergen Ara h1 using a stem-loop DNA biosensor modified with a chitosan-mutiwalled carbon nanotube nanocomposite and spongy gold film. *Talanta* **131**, 521-527 (2015).
15. Chatelain, G., Ripert, M., Farre, C., Ansanay-Alex, S. & Chaix, C. A “four-ferrocene” modified stem-loop structure as a probe for sensitive detection and single-base mismatch discrimination of DNA. *Electrochim. Acta* **59***,* 57-63 (2012).
